# Total-Factor Eco-Efficiency and Its Influencing Factors in the Yangtze River Delta Urban Agglomeration, China

**DOI:** 10.3390/ijerph16203814

**Published:** 2019-10-10

**Authors:** Yongyi Cheng, Tianyuan Shao, Huilin Lai, Manhong Shen, Yi Li

**Affiliations:** 1 School of Business, Ningbo University, Ningbo 315211, China; styka1@163.com (T.S.); laihuilin1125@outlook.com (H.L.); smh@nbu.edu.cn (M.S.); 2 Center for Ecological Civilization of Yangtze River Delta, Ningbo University, Ningbo 315211, China; 3 East China Sea Institute, Ningbo University, Ningbo 315211, China; 4 International United Faculty between Ningbo University and University of Angers, Ningbo University, Ningbo 315211, China

**Keywords:** sustainable development, urban, eco-efficiency, influencing factors, SBM, DEA, spatial panel model, Tobit model, Yangtze River Delta Urban Agglomeration

## Abstract

Urban agglomerations are not only the core areas leading economic growth but also the fronts facing severe resource and environmental challenges. This paper aimed to increase our understanding of urban eco-efficiency and its influencing factors and thus provide the scientific basis for green development. We developed a model that incorporates super-efficiency, slacks-based-measure, and global-frontier technology to calculate the total-factor eco-efficiency (TFEE) and used a spatial panel Tobit model to take into account spatial spillover effects. An empirical study was conducted utilizing a prefecture-level dataset in the Yangtze River Delta Urban Agglomeration (YRDUA) from 2003 to 2016. The main findings reveal that significant spatial differences exist in TFEE in the YRDUA: high-TFEE cities were majorly located in the coastal areas, while low-TFEE cities were mostly situated inland. Overall, TFEE shows a trend of “decline first and then rise with fluctuation”; the disparity between inland and coastal regions has expanded. Further regression analysis suggests that industrial structure, environmental regulation, and innovation were positively related to TFEE, while foreign direct investment was not conducive to the growth in TFEE. The relationship between population intensity and urban eco-efficiency is an inverted U-shaped curve. Finally, several specific policy implications were raised based on the results.

## 1. Introduction

As environmental pollution and resource crises intensified, policymakers are increasingly concerned about the impact of economic activities on the ecological environment. Urban agglomerations are not only the core areas leading economic growth but also the fronts facing severe resource and environmental challenges. The spatial differentiation of economic development and of ecological environment exist simultaneously within urban agglomerations. To promote green and coordinated development, it is urgent to improve the performance appraisal system of regional development and to optimize the interregional cooperation mechanism. Eco-efficiency, which takes into account factors such as resources, environment, and economy, can reflect green development performance comprehensively, and therefore, providing an important reference for measuring the long-term development advantages of a region and formulating sustainable development policies. Undoubtedly, the calculation of urban eco-efficiency and the identification of its influencing factors are prerequisites for achieving sustainable urban development [1].

The concept of eco-efficiency was first introduced by Schaltegger and Sturm [2] and has attracted much attention since [3]. The World Business Council for Sustainable Development (WBCSD) defines eco-efficiency as providing cost-effective services and products to meet human needs and high-quality life; at the same time, these services and products can reduce environmental impacts and resource consumption to levels that match the Earth’s carrying capacity [4]. According to Kuosmanen [5], eco-efficiency emphasizes acquiring economic output with minimal natural resources consumption and environmental degradation. Since eco-efficiency essentially reflects the effectiveness of input and output, it is suitable for the application of frontier analysis methods, which mainly including the Data Envelopment Analysis (DEA) method [6] and the Stochastic Frontier Analysis (SFA) method. Compared with the SFA model, the DEA model provides a more straightforward and more flexible estimation method, as there is no need for knowledge of the functional relationship between input and output, let alone assigning input and output weights [7], which is why it has gained more popularity.

Various models have been developed to evaluate eco-efficiency. For instance, Korhonen [8] estimated the eco-efficiency of 24 European power plants using an extended DEA model that treats pollutants as the inputs. Sarkis [9] provided six DEA models and some extensions to assess the ecological efficiency of power plants. Zhang et al. [10] took six kinds of environmental pollutant emissions together with water resources, raw mining resources, and energy as the inputs of DEA to measure ecological efficiency. However, as shown in many studies, conventional DEA models have some drawbacks. An eco-efficiency derived from a contemporaneous production possibility set (PPS) may encounters problems of misleading technical regress and also suffers non-circularity and linear programming infeasibility when measuring cross-period directional distance functions (DDF), which are referred to as “discriminating power problem” and “technical regress” [11,12]. To overcome these problems, scholars have developed many improved approaches. Tone [13,14] put forward the super-efficiency model based on the non-angular, non-radial Slacks-based Measure (SBM) model to address the “discriminating power problem”. Additionally, Pastor and Lovell [15] proposed a new model based on global benchmark technology. Oh [16] further extended it with sequential technology, which is circular and can overcome “technical regress”, and thus, has been widely used in the estimation of eco-efficiency in recent years [17]. For example, using an improved SBM model combines global benchmark technology, directional distance function and a bootstrapping method with the Global Malmquist Luenberger (GML) index, Yang and Zhang [18] analyzed the dynamic trends of eco-efficiency of 30 sample provinces in mainland China from 2003 to 2014. Adopting the SBM model, Zheng et al. [19] measured the eco-efficiency of 31 Chinese regions from 2000 to 2015. Furthermore, based on a Meta-US-SBM model (meta-frontier, undesirable outputs, and super-efficiency SBM), Huang et al. [20] estimated the composite eco-efficiency using in China’s provincial data from 2001 to 2014.

Some studies have explored the factors that influence eco-efficiency. For example, Zhang et al. [21] examined the factors affecting industrial eco-efficiency based on the three-stage DEA model using Chinese provincial data between 2005 and 2013, and their results showed that China’s regional industrial eco-efficiency was majorly affected by the factors of the environmental regulations, technological innovations, as well as level of economic development and industrial structure. Bai et al. [22] quantitatively investigated the relationship between urbanization and urban eco-efficiency using the data of 281 prefecture-level cities in China from 2006 to 2013. Their results showed that apparent spatial disparities exist among different cities, and there was an N-shaped relationship between urbanization and urban eco-efficiency. Ren et al. [23] analyzed the effects of three types of environmental regulations, namely, command-and-control, market-based, and voluntary regulation, on the eco-efficiency of China’s three regions based on the Stochastic Impacts by Regression on Population, Affluence and Technology (STIRPAT) model. Their findings revealed that the effects of different types of environmental regulation on eco-efficiency vary from region to region. Huang et al. [24] investigated the impacts and mechanisms of the urban cluster on urban eco-efficiency. They find that the improvement of the urban cluster is conducive to enhancing urban eco-efficiency, and there is a “core-periphery” spatial structure in the process of urban cluster development. Li et al. [25] studied the relationship between government transparency and eco-efficiency utilizing the data of 262 cities in China from 2005 to 2012. Their results suggested that the overall eco-efficiency of Chinese cities was low, and a nonlinear relationship exists between government transparency and eco-efficiency performance.

However, despite all the fruitful results and substantial advances, there are still some limitations in the previous literature. Firstly, compared with numerous studies that focused on the provincial and sectoral level [21], much less attention has been paid to the eco-efficiency of cities, especially that of the emerging cities in developing countries [26]. Considering that urban areas make a tremendous contribution to resource consumption and pollution emissions in the developing world, it is of great significance to carry out in-depth research on their green development. Another essential drawback is that most studies ignored the spatial correlations between cities when discussing the influencing factors of urban eco-efficiency. However, apparent neglect of the spatial spillover effects, which tend to become significant with the intensification of interregional economic connections and the more frequent flow of factors of production, could result in biased estimations [1,27].

To address these limitations of extant studies, this paper extends research at the provincial and sectoral level to the urban level utilizing a prefecture-level panel dataset in the Yangtze River Delta Urban Agglomeration (YRDUA), China, during the period of 2003–2016, and constructs a model that incorporated the super-efficiency DEA model with slacks-based-measure and global-frontier technology (SSBM-GF) to estimate total-factor eco-efficiency (TFEE). Moreover, to take into account spatial spillover effects, a spatial panel Tobit model is constructed to analyze the influencing factors of urban eco-efficiency. The results are supportive of understanding the spatial difference and driving mechanism of urban eco-efficiency in the YRDUA, providing a scientific basis for governments to formulate policies to promote the development of green and sustainable urbanization.

The remainder of the paper is organized as follows. Section 2 introduces the methodology and data sources. Section 3 presents the empirical results. Section 4 provides some discussion and implications. Section 5 provides a conclusion.

## 2. Methodology and Data

### 2.1. Super-Efficiency Slacks-Based Measure Global Frontier Model

Following Fare et al. [28], the non-parametric Data Envelopment Analysis (DEA) piecewise linear production frontiers are adopted in this study to estimate total-factor eco-efficiency. To overcome the “discriminating power problem” [11,12], Tone [13,14] proposed the super-efficiency model based on the non-radial, non-angular Slacks-based Measure (SBM) model. Furthermore, Pastor and Lovell [15] introduced a global benchmark technology to address the “technical regress” problem [11,12]. Thus, to ensure that the calculations were accurate, we combined the super-efficiency SBM model with global frontier technology to construct a Super-efficiency Slacks-based Measure Global Frontier (SSBM-GF) model.

Given that x represents each of the M inputs of a decision-making unit (DMU, that is, the cities in this study) such that $x\;=\;\left(x_{1}\cdots x_{M}\right)\in R_{M}^{+}$, y represents each of the S desirable outputs of the DMU such that $y\;=\;\left(y_{1}\cdots y_{S}\right)\in R_{S}^{+}$, b represents each of the V undesirable outputs of the DMU such that $b\;=\;\left(b_{1}\cdots b_{V}\right)\in R_{V}^{+}$, $\left(x_{i},y_{i},b_{i}\right)$ is the vector for the inputs, desirable outputs, and undesirable outputs of DMU_i_ (i = 1 ... N), and $\left(s_{x},s_{y},s_{b}\right)$ is the slacks vector, then a production possibility set (PPS) can be expressed by:

(1) $$PPS\;=\;\left\{\left(x_{i},y_{i},b_{i}\right)|x_{i}\geq \sum_{i\;=1}^{N}x_{i}\lambda_{i},y_{i}\leq \sum_{i=1}^{N}y_{i}\lambda_{i},\;b_{i}\geq \sum_{i=1}^{N}b_{i}\lambda_{i},\lambda_{i}\geq 0\right\}$$

where λ is an unknown weight vector.

Thus, the PPS of the global frontier is given by:

(2) $$PPS^{Glb}\;=\;\left\{PPS^{1}\cup^{\;}PPS^{2}\cup^{\;}\ldots \cup^{\;}PPS^{T}\right\}$$

where $PPS^{Glb}$ denotes the specific technologies of the global frontier (i.e., best practice frontier) [29]. The production technology is assumed to follow all the standard axioms of production theory, including the assumptions of bounded set, bounded convexity, etc. [30,31].

To calculate the super-efficiency of a specific DMU, PPS is constructed by eliminating the observations of that specific DMU. The non-radial distance function is used to measure the efficiency gap between the production frontier and a certain specific DMU. Hence, the TFEE, which measures the distance of observed DMU_0_ from the global frontier, can be calculated by solving the linear programming (LP) problem given below:

(3) $$\mathrm{TFEE}\;=\;\min\left\{\frac{1-\frac{1}{2}\left(\frac{1}{M}\sum_{j\;=\;1}^{M}\frac{s_{0j}^{x}}{x_{0j}}+\frac{1}{V}\sum_{k\;=\;1}^{V}\frac{s_{0k}^{b}}{b_{0k}}\right)}{1+\frac{1}{S}\sum_{l\;=\;1}^{S}\frac{s_{0l}^{y}}{y_{0l}}}\right\},\;s\;.t.\;\;\{\begin{array}{c} x_{0}\;=\;\underset{i\neq 0}{\sum }\left(x_{i}\lambda_{i}\right)\;+\;s_{0}^{x}\\ b_{0}\;=\;\underset{i\neq 0}{\sum }\left(b_{i}\lambda_{i}\right)\;+\;s_{0}^{b}\\ y_{0}\leq \underset{i\neq 0}{\sum }\left(y_{i}\lambda_{i}\right)\\ s_{0}^{x}\geq 0,s_{0}^{b}\geq 0,\lambda \geq 0,\overset{N}{\underset{i\;=\;1}{\sum }}\lambda_{i}\;=\;1 \end{array},$$

where $s^{x}$, $s^{,b}$, and $s^{y}$ denote the slack in input, desirable output, and undesirable output, respectively.

### 2.2. Spatial Panel Tobit Model

Since TFEE, the dependent variable in our econometric model, always has a value that is no less than 0, it is not suitable to use the ordinary least squares (OLS) for coefficient estimation. Otherwise, it would lead to a problem in terms of the inconsistency of coefficient and biased estimates. On that account, the Tobit model [32], which has been widely used to investigate the influencing factors of environmental efficiency [33], was adopted in this paper.

Additionally, economic activities in one city generally exert a spillover effect on neighboring cities [34]. To take into account the spatial spillover effects of TFEE, a spatial panel Tobit model was constructed in this study. The spatial lag model and the spatial error model are widely used in spatial econometric modeling. The spatial lag Tobit model can be expressed by:

(4) $$TFEE_{it}\;=\;max\;\left(0,\alpha +\rho WTFEE_{it}+\beta X_{it}+\varepsilon_{it}\right)$$

where $TFEE_{it}$ is the total-factor eco-efficiency, α is the constant term; ρ is the spatial lag parameter, W is the spatial weights matrix, β is the coefficient of the explanatory variables, $X_{it}$ stands for the explanatory variables, and $\varepsilon_{it}$ is the error term.

The spatial error Tobit model can be expressed by:

(5) $$TFEE_{it}\;=\;max\left(0,\alpha_{i}+\beta X_{it}+\mu_{it}\right)\;\mu_{it}\;=\;\theta WTFEE_{it}+\varepsilon_{it}$$

where θ is the spatial autocorrelation coefficient. As for how to select between the above two models, Anselin et al. [35] suggested the use of the Lagrange multiplier (LM-lag and LM-error) and its robust form (Robust LM-lag and Robust LM-error).

The spatial weights matrix W was constructed based on geographical distances due to the consideration that, compared with the spatial adjacency matrix, the distance weights matrix is more frequently employed, as it can reflect the attenuation characteristics of spatial spillover. The matrix element $w_{ij}$ is defined as:

(6) $$w_{ij}\;=\;\{\begin{array}{l} \frac{1}{d_{ij}}\;,\;i\neq j\\ \;0\;,\;i\;=\;j \end{array}$$

where $d_{ij}$ is the distance between cities *i* and *j*.

### 2.3. Variables Selection

The calculation of TFEE integrated three dimensions: the economy, resources, and the environment:

(1) Inputs. The inputs include capital, labor, energy, and water consumption. Capital input (*K*): the capital stock is estimated by adopting the Perpetual Inventory Method [36,37]. Labor force (*L*): the average number of employees each year. Energy (*E*): following extant literature, we used the amount of electricity consumption as a proxy for energy input due to the lack of data on final energy consumption at the city level [38]. Water (*W*): the amount of water consumption.
(2) Undesirable outputs. The undesirable outputs consist of three types of pollutants, namely, Wastewater (*WW*), sulfur dioxide (*SO2*), and soot and dust (*SD*).
(3) Desirable output. Gross domestic product (GDP) is defined as the desirable output of each city.

To investigate the determinants of total-factor eco-efficiency, we specify an econometric model. Based on previous research in this field, the following factors were included as the explanatory variables in this study:

(1) Industrial structure (*IS*). Urban eco-efficiency can be affected by the industrial structure. Compared with the secondary industry, the tertiary sector is much less relying on resources and creates fewer pollutants; therefore, a higher ratio of the service sector may lead to lower pressure on the urban environment, which can, in turn, lead to a better eco-efficiency. Thus, we use the tertiary industry ratio as an indicator to represent the industrial structure.
(2) Environmental regulations (*ER*). Environmental regulations are critical for resource-saving and pollution control, as well as the promotion of green development [39]. Compared with the operating costs of pollution control facilities, the income level is a better proxy for environmental regulation. The main reason is that using the former indicator may encounter significant endogenous problems in the econometric analysis since there is an apparent two-way causality; that is, not only operating costs of pollution control facilities can affect environmental quality, but also the level of pollution will determine the expenditure. By contrast, there is no such problem with the latter one. As the income level rises, the citizen’s demand for a better environment will also increase, thus urging the government to improve environmental governance properly. Following Antweiler et al. [40], we used GDP per capita as a proxy for environmental regulation.
(3) Innovation (*INN*). Innovation plays a crucial role in improving environmental performance [41]. Above all, innovation can promote the improvement of production technology, and therefore, can reduce the input of raw materials and energy consumption of unit products. In addition, innovation can also give impetus to the emerging industries and become a new driving force for the economy [42]. The ratio of scientific expenditure to total fiscal spending is used to define a city’s innovation intensity.
(4) Foreign direct investment (*FDI*). The relationship between FDI and green development is uncertain. The advanced technologies that come with FDI could help to promote economic development, but resource consumption and pollution emission might also increase as a result of FDI and thus have a negative impact on eco-efficiency [43,44,45,46,47,48,49]. We use the ratio of real FDI to real GDP to measure FDI inflows.
(5) Population density (*PD*). There is also an undetermined relationship exists between population density (the ratio of population to the built-up area) and urban eco-efficiency. Cropper and Griffiths [50] pointed out that higher population density may lead to higher pressure on the environment, which can, in turn, lead to a decrease in TFEE. However, Liu et al. [51] suggested that higher population density may urge society to pay more attention to the environment, and it may improve TFEE. Therefore, Population density and its squared term (*PD2*) are also included in our model to test whether there was an environmental Kuznets curve (EKC) for TFEE and population density.

### 2.4. Study Area and Data Sources

**Figure 1:**
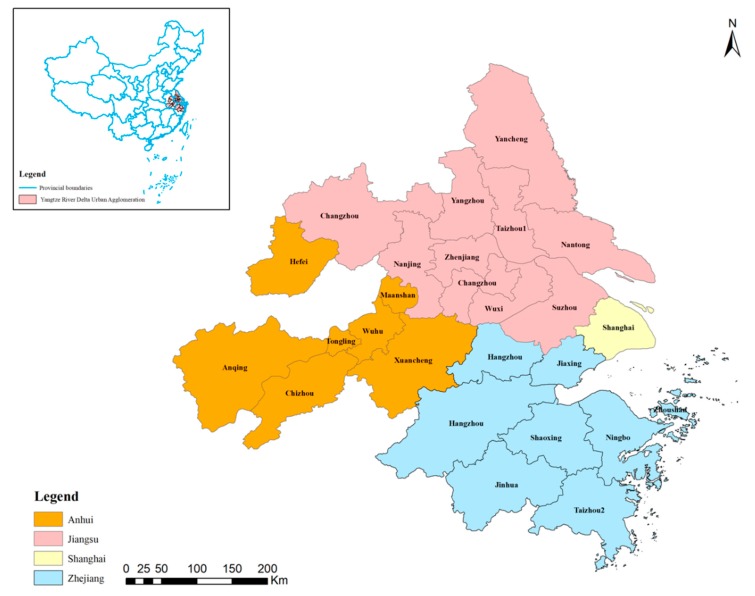
Study area.

The Yangtze River Delta Urban Agglomeration is one of the six largest metropolitan areas in the world and the largest metropolitan area in China [52]. The YRDUA mainly consists of Shanghai City, Jiangsu Province, Zhejiang Province, and Anhui Province. This study included 26 prefecture-level cities of the YRDUA, among which nine cities are located in Jiangsu Province, eight cities are located in Zhejiang Province, and the other eight cities are located in Anhui Province (see Figure 1).

The study period covered the years 2003–2016, and the data were collected from the *Chinese City Statistical Yearbook* [53], the *China Statistical Yearbook on Environment* [54], and the *Annual Statistical Report on Environment in China* [55]. All monetary variables are adjusted to 2000 constant prices using the corresponding price indices. Table 1 lists the descriptive statistics for the relevant variables.

## 3. Empirical Results

### 3.1. Analysis of TFEE

The TFEE of cities in the YRDUA during 2003–2016 were evaluated by solving Equation (3), and the results are analyzed in two dimensions: spatial distribution and temporal evolution.

#### 3.1.1. Spatial Distribution of TFEE

**Figure 2:**
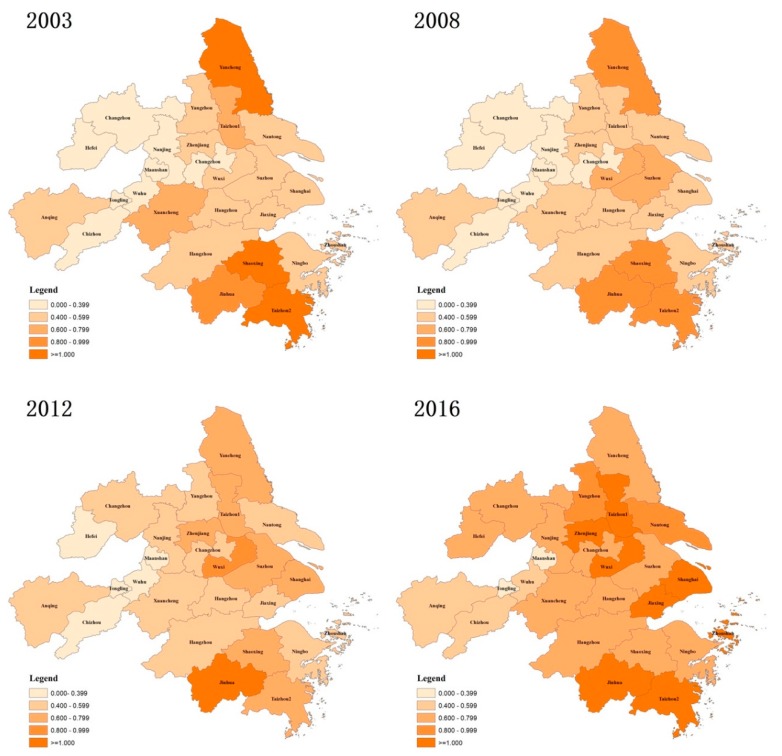
Spatial distribution of total-factor eco-efficiency (TFEE) in the Yangtze River Delta Urban Agglomeration (YRDUA), 2003–2016.

As shown in Figure 2, significant spatial differences existed in TFEE in the YRDUA. The cities with high eco-efficiency were majorly located in the coastal areas, while the cities with low eco-efficiency were mostly situated in the inland parts throughout the study period. Moreover, the number of cities with high eco-efficiency shrank during the period 2003–2012, and since then has increased significantly. Specifically, there were four cities with TFEE values exceeding 0.8 in the year 2003 and 2008, which were Yancheng city in Jiangsu Province and Shaoxing, Jinhua and Taizhou2 city in Zhejiang Province. However, there was only one city with TFEE values exceeding 0.8 in the year of 2012, which was Jinhua city in Zhejiang Province. In the year of 2016, nine cities were found to be with TFEE values exceeding 0.8, but none of them was located in the inland Province, Anhui.

#### 3.1.2. Temporal Evolution of TFEE

**Figure 3:**
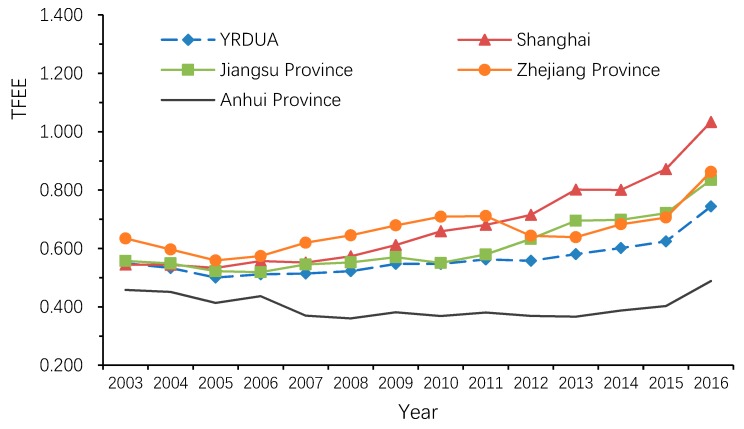
TFEE in the YRDUA and its four regions, 2003–2016.

As demonstrated in Figure 3, TFEE in the YRDUA and its four regions showed a similar trend of “decline first and then rise with fluctuation” during the period 2003–2016. More specifically, TFEE in the YRDUA as a whole declined obviously during the period 2003–2005, and since then has remained almost unchanged during 2005–2012, and finally increased gradually during the period 2012–2016. In addition, among the four regions in the YRDUA, Shanghai and Jiangsu Province enjoyed the most stable growth of TFEE than the other two areas. Notably, TFEE in Anhui Province was the lowest among four regions in the YRDUA, and the gap between Anhui Province and the other regions has widened during the study period due to a long-time stagnation of its TFEE.

### 3.2. Influencing Factors of Total-Factor Eco-Efficiency

Stata 14.0 software was used to estimate the spatial econometric models in this study, and variables measuring by non-percentage indicators were transformed to logarithms in the model to reduce the degree of dispersion. In order to explore the differences between areas and to test the robustness of the estimation results, we divided the 26 cities into high-income group, which mainly included the coastal cities, and the low-income group, which primarily included the inland cities, according to GDP per capita in the year of 2003. Then, we conducted an econometric analysis using the full sample and the two subsamples, respectively. Moreover, before estimating the coefficients of all samples, we firstly tested the spatial correlation based on the residuals of the corresponding non-spatial OLS estimations. The test results in Table 2 provide two essential information: (1) the values of Moran’s I are greater than zero and significant at a 5% level in all samples, indicating that the TFEE of the cities has a positive spatial autocorrelation; (2) the strong statistical significance of the LM-lag tests suggest that the spatial lag models are suitable in all samples, while some of the LM-err statistics are insignificant. Therefore, the spatial lag Tobit model was selected rather than the spatial error Tobit model. Table 3 reports the estimated results of the spatial lag Tobit model assuming both fixed and random effects.

The coefficients for spatial autoregressive terms, W*TFEE, are positive and significant, indicating a clear spatial spillover effect of eco-efficiency across cities in the YRDUA. A comparison between the estimation results for the high-income group and low-income group suggests that the spatial spillover effect of eco-efficiency was more evident in the low-income cities than in the high-income cities. This difference could be explained by the following reasons to a certain extent: low-income cities tend to have low eco-efficiencies fundamentally due to their production inefficiency; however, quite a few high-income cities were considerably eco-inefficient because of their environmental inefficiency.

The coefficients for industrial structure, IS, are positive and significant at the 5% level, suggesting that an increase in the proportion of tertiary industry could promote the improvement of eco-efficiency in the YRDUA. This result is consistent with the theory and previous studies. Changes in the industrial structure will result in changes in intensities of energy consumption and pollution emission, and will consequently have great effects on the environment. It is asserted that the difference in industry structure may inevitably affect the level of eco-efficiency [46]. In addition, the effects of industrial structural change on urban eco-efficiency varied by income level. The effect of variation of industrial structure in low-income cities was greater than that in the high-income cities.

The coefficients for environmental regulation, ER, are significantly positive, implying that the impact of environmental regulation on eco-efficiency was quite positive in the YRDUA. This outcome is similar to Wang et al. [42]. For the cities of the YRDUA, strict environmental regulations can promote both economic prosperity and environmental quality and therefore lead to a win-win situation. Hence, the Porter hypothesis [27,28] is supported by this study. Moreover, the effects of environmental regulation on urban eco-efficiency also varied by income level. The effect of environmental regulation in the high-income cities was greater than that in low-income cities. Therefore, the government should take measures to upgrade environmental supervision and increase environmental investment in backward cities.

The coefficients for innovation intensity, INN, are positive and significant at the 1% level, suggesting that the enhancement of innovation intensities has a positive effect on eco-efficiency. Innovation is an essential driving force for sustained economic growth and the key to maintaining the core competitiveness of the industry [47]. Technological innovation can promote the development of green technologies such as energy-saving and emission cutting [36], and therefore, improve environmental quality by reducing resource consumption and pollution emission.

The coefficients for FDI are negative but not significant in the YRDUA, suggesting that FDI has not promoted the growth in eco-efficiency in this region. This result is similar to Wen [29], who suggested that the impacts of FDI on total productivity differed by region in China. The reason may be that foreign investment mainly focused on manufacturing, consequently bringing about tremendous pressure on the resources and environment in the YRDUA. Although “pollution transfer” may occur during the inflow of FDI, the advanced technology and management experience coming with the transfer process was also conducive to improving the environmental quality of the host country [52].

The coefficients for population intensity, PD, are positive and significant in all samples, while their squared terms, PD2, illustrating a significant (10% level) negative sign only in the high-income cities. This implies that the relationship between population intensity and eco-efficiency is an inverted U-shaped curve. That is, below some critical value, the increase in urban population intensity can promote eco-efficiency; otherwise, it may harm the sustainability of urban areas.

## 4. Discussion and Implications

This study shows that there were significant regional disparities of TFEE in the YRDUA. The cities with high eco-efficiency were majorly located in the coastal areas, while the cities with low eco-efficiency were mostly situated in the inland parts throughout the study period. This finding is similar to Xing et al. [56], which suggests that these spatial disparities are primarily attributable to geographical and economic differences among areas. Compared with the cities in the inland areas, the cities in the coastal areas are more developed. They have better infrastructure, more advanced technologies and more stringent environmental regulation, which contribute to improving resource utilization and reducing pollutant emissions and thus promoting urban eco-efficiency.

TFEE in the YRDUA and its four regions showed a similar trend of “decline first and then rise with fluctuation” during the period 2003–2016. The temporal evolution of TFEE in the YRDUA generally reflected the transformation of China’s economic development model during this period. After joining the WTO in 2001, China started a process of rapid industrialization. As the frontier for opening up to the outside world, the Yangtze River Delta quickly developed a thriving manufacturing industry during this period, and consequently put enormous pressure on resources and environment. Therefore, TFEE in the YRDUA showed a downward trend during 2003–2005. In response to the deteriorating ecological environment, the Chinese central government introduced a series of regulatory policies and increased environmental governance. For instance, Export Control of High-energy-consuming, High-polluting and Resource-intensive Products (2005), Assessment of Corporate Environmental Behavior (2005), Government Procurement List of Energy-saving Products (2006), Emissions Trading Scheme (2006), Environmental Liability Insurance System (2007), Project for Reducing Major Pollutants Emission (2007), Energy Conservation Law (2008), etc. However, stimulated by the blooming external demand before the global financial crisis, China’s economy was heavily reliant on resources, presenting a characteristic of high pollution and high growth. Thus, economic growth at this stage was still not coordinated with environmental improvement in the YRDUA; thus, TFEE in this area showed a fluctuating state during the period 2005–2012. In the post-financial crisis era, China gradually strengthened ecological protection and environmental governance and proposed an “ecological civilization construction” strategy. As the most developed region in China, the Yangtze River Delta has taken the lead in ecological construction by accelerating the elimination of low-end industries and encouraging innovation, which has significantly reduced pollution and improved resource utilization efficiency. Accordingly, a steady increase of TFEE in the YRDUA has been observed during the period 2012–2016.

Therefore, some policy implications for achieving green and sustainable development in urban areas can be put forward based on this research. Firstly, the pace of integrated development of the Yangtze River Delta Urban Agglomeration should be accelerated. There was an apparent disparity of ecological efficiency among cities in the Yangtze River Delta, and the gap between inland and coastal cities has been expanding, which is not conducive to the construction of regional ecological civilization. To achieve coordinated and shared development, it is urgent to strengthen guidance and support for the backward areas and give full play to positive spillover effects. While strengthening the responsibility for ecological protection, it is also indispensable to promoting the free flow of factors of production and encouraging the dispersion of advanced technologies and industries to backward areas, thereby gradually eliminate regional inequality.

Secondly, the improvement of ecological efficiency should be set as one of the core factors in the performance assessment system for local government. In a decade (2003–2012), the ecological efficiency of the Yangtze River Delta urban agglomeration has been stagnated. The fundamental reason is that the old development model places too much emphasis on GDP, thus neglecting resources, environment, and ecology. In recent years, especially after 2012, the reform of the performance assessment system for local government has had a profound impact on the resources and environment. The central government, whose incentives play a vital role in shaping regional plans [57], has firmly strengthened the supervision of the ecological environment, placing more focus on the preservation of natural resources and the improvement of environmental quality. Accordingly, the urban eco-efficiency of the Yangtze River Delta region has achieved steady growth since 2012. However, Chinese cities still have a lot of room to improve their eco-efficiency, which depends to a great extent on further reforming the performance evaluation mechanisms to improve green development.

Thirdly, a timely enhancement of environmental regulations is critical for developing economies to achieve high-quality development. As industrialization goes to a certain stage, developing economies should raise their environmental standards promptly, and put a limit on the energy-intensive and high-pollution industries, and therefore avoid becoming the “pollution heaven” for FDI. For the Yangtze River Delta Urban Agglomeration, more stringent environmental regulations are needed in the future to formulate and implement regional integrated environmental policies.

Furthermore, steady investing in innovation is indispensable to promoting eco-efficiency. Innovation is vital to the development of emerging industries and is the fundamental driving force for achieving sustainable development. Therefore, the government must continuously invest financial resources to support scientific research, technology development and business innovation in multiple dimensions, and create an efficient innovation system. Furthermore, the increase in the proportion of the tertiary industry can reduce resource consumption and pollution emissions, thereby improving ecological efficiency. Hence, the government should emphasize the transformation and upgrading of industries in the backward cities and encourage the development of service industries.

Finally, population concentration should be well-guided to make full use of scale effects and agglomeration effects. There was a weak inverted U-type relationship between population density and eco-efficiency in the YRDUA; thus, differentiated urbanization policies should be formulated for cities of different sizes. For small and medium-sized cities, population concentration should be further enhanced to make full use of scale effects and agglomeration effects to improve economic efficiency and environmental efficiency. However, for some big cities such as Shanghai, Hangzhou, and Nanjing, properly control of the population density is essential to prevent “big city diseases” from threatening sustainable development.

## 5. Conclusions

It is of great significance to carry out in-depth research on the dynamics and driving mechanisms of eco-efficiency of urban areas in developing countries since they make a tremendous contribution to resource consumption and pollution emissions in the developing world. This paper extends research at the provincial and sectoral level to the urban level utilizing a prefecture-level panel dataset in the Yangtze River Delta Urban Agglomeration, China, between 2003 and 2016, and proposes an SSBM-GF model that incorporated the super-efficiency DEA model with slack-based-measure as well as global-frontier technology to estimate total-factor eco-efficiency. Moreover, to take into account spatial spillover effects, a spatial lag Tobit model is constructed to analyze the factors influencing urban eco-efficiency.

Our measure revealed that there were great regional disparities of TFEE in the YRDUA; the cities with high eco-efficiency were majorly located in the coastal areas, while the cities with low eco-efficiency were mostly situated in the inland areas throughout the study period. TFEE in the YRDUA and its four regions demonstrated a similar trend of “decline first and then rise with fluctuation” during the period 2003–2016. The regression analysis shows that there was a noticeable positive spatial spillover effect of eco-efficiency across cities in the YRDUA. An increase in the proportion of tertiary industry could promote the improvement of eco-efficiency. Moreover, the impacts of environmental regulation and innovation on eco-efficiency both were significantly positive in the YRDUA. Notably, the inflow of FDI was not conducive to the growth in eco-efficiency in this region. The relationship between population intensity and eco-efficiency is an inverted U-shaped curve. The results are supportive of understanding the spatial difference and driving mechanism of urban eco-efficiency in the YRDUA, providing a scientific basis for governments to formulate policies to promote the development of green and sustainable urbanization.

This study inevitably has some limitations, which in turn point to directions for future research. Firstly, this paper used an SSBM-GF model based on the DEA method to estimate the total-factor eco-efficiency. Although DEA has some advantages, it is a non-parametric mathematical programming approach that does not consider statistical noise, which might lead to biased measures to a certain extent. Moreover, our analysis merely focused on the Yangtze River Delta region; therefore, the findings should not be taken as an accurate depiction of the overall picture of the urban development across China. Nevertheless, this approach can be extended to more parts of China and other countries without difficulty.

## Figures and Tables

**Table 1:** Statistics for the relevant variables.

Input/Output	Variable	Units	Mean	Standard Deviation	Max	Min	Observations
Inputs	Capital	100 million RMB	7277.30	7500.36	36,127.26	153.58	364
	Labor	10 Thousand People	325.73	214.61	1368.91	41.66	364
	Energy	tens of MW h	1,530,223.00	2,423,116.12	14,860,200	43,836	364
	Water	10 Thousand Mt	34,462.26	61,688.60	346,068	1500	364
Undesirable Outputs	Wastewater	10 Thousand Mt	17,437.83	18,055.32	85,735	596	364
	Sulfur dioxide	Thousand Mt	65.27	64.08	496.378	1.93	364
	Soot	Thousand Mt	32.40	23.64	141.73	1.25	364
Desirable Outputs	Gross domestic product	100 million RMB	2421.31	3058.77	22,195.93	73.00	364

**Table 2:** Tests of residual spatial correlation based on ordinary least squares (OLS) estimation results.

Variable	Full Sample	Low-Income Group	High-Income Group
Moran’s I	0.752 **	0.805 **	0.693 **
LM-lag	11.996 **	14.210 ***	9.872 **
LM-error	2.828 *	4.093 *	1.657

Note: Moran’s I represents Moran’s index, LM-lag represents the statistic of the Lagrange Multiplier test for spatial lag dependence, LM-error represents the statistic of the Lagrange Multiplier test for spatial error dependence; ***, **, and * denote significance levels of 0.01, 0.05, and 0.10, respectively.

**Table 3:** Estimated determinants of total-factor eco-efficiency.

Variables	Full Sample		Low-Income Group		High-Income Group	
	(1) Fixed Effects	(2) Random Effects	(3) Fixed Effects	(4) Random Effects	(5) Fixed Effects	(6) Random Effects
W*TFEE	0.137 ** (0.077)	0.151 ** (0.082)	0.146 ** (0.071)	0.154 ** (0.076)	0.129 * (0.080)	0.147 * (0.092)
IS	0.257 *** (0.101)	0.297 *** (0.095)	0.318 *** (0.084)	0.385 ** (0.202)	0.185 ** (0.097)	0.206 ** (0.113)
ER	0.087 *** (0.044)	0.091 *** (0.024)	0.064 ** (0.035)	0.080 ** (0.042)	0.101 *** (0.042)	0.103 *** (0.029)
INN	0.064 *** (0.016)	0.060*** (0.019)	0.059 *** (0.015)	0.064 *** (0.011)	0.068 *** (0.019)	0.052 *** (0.016)
FDI	−0.015 (0.013)	−0.010 * (0.006)	−0.013 (0.029)	−0.009 (0.007)	−0.016 (0.015)	−0.011 * (0.006)
PD	0.069 * (0.040)	0.084** (0.034)	0.043 * (0.023)	0.050 ** (0.029)	0.087 * (0.053)	0.117 * (0.059)
PD^2^	−0.009 (0.022)	−0.005 (0.015)	−0.012 (0.030)	−0.009 (0.011)	−0.007 * (0.004)	−0.009 * (0.005)
Log-likelihood	310.601	290.223	152.031	156.582	140.725	145.248
Observations	364	364	182	182	182	182

Note: W is the spatial weights matrix. The values in parentheses are the standard deviations corresponding to the respective estimated parameters; ***, **, and * denote significance levels of 0.01, 0.05, and 0.10, respectively.
